# *In Vitro* Antiosteoporosis Activity and Hepatotoxicity Evaluation in Zebrafish Larvae of Bark Extracts of *Prunus jamasakura* Medicinal Plant

**DOI:** 10.1155/2020/8582318

**Published:** 2020-09-15

**Authors:** Richard Komakech, Ki-Shuk Shim, Nam-Hui Yim, Jun Ho Song, Sun Kyu Yang, Goya Choi, Jun Lee, Yong-goo Kim, Francis Omujal, Moses Agwaya, Grace Kyeyune Nambatya, Hyemin Kan, Kyu-Seok Hwang, Gilbert Matsabisa Motlalepula, Youngmin Kang

**Affiliations:** ^1^Herbal Medicine Resources Research Center, Korea Institute of Oriental Medicine (KIOM), 111 Geonjae-ro, Naju-si, Jeollanam-do 58245, Republic of Korea; ^2^University of Science & Technology (UST), Korean Convergence Medicine Major, KIOM, 1672 Yuseongdae-ro, Yuseong-gu, Daejeon 34054, Republic of Korea; ^3^Natural Chemotherapeutics Research Institute (NCRI), Ministry of Health, P.O. Box 4864, Kampala, Uganda; ^4^Korea Institute of Oriental Medicine (KIOM), 1672 Yuseongdae-ro, Yuseong-gu, Daejeon 34054, Republic of Korea; ^5^Korean Medicine Application Center, Korea Institute of Oriental Medicine, 70 Cheomdan-ro, Dong-gu, Daegu 41062, Republic of Korea; ^6^Bio & Drug Discovery Division, Korea Research Institute of Chemical Technology, Daejeon, Republic of Korea; ^7^IKS Research Group, Department of Pharmacology, Faculty of Health Sciences, University of the Free State, Bloemfontein 9301, Free State, South Africa

## Abstract

Osteoporosis is one of the main health problems in the world today characterized by low bone mass and deterioration in bone microarchitecture. In recent years, the use of natural products approach to treat it has been in the increase. In this study, *in vitro* antiosteoporosis activity and hepatotoxicity of *P*. *jamasakura* bark extracts were evaluated. *Methods*. Mouse bone marrow macrophage (BMM) cells were incubated with tartrate-resistant acid phosphate (TRAP) buffers and *p*-nitrophenyl phosphate and cultured with different *P*. *jamasakura* bark extracts at concentrations of 0, 6.25, 12.5, 25, and 50 *μ*g/ml in the presence of the receptor activator of nuclear factor kappa-Β ligand (RANKL) for 6 days. The osteoclast TRAP activity and cell viability were measured. Nitric oxide (NO) assay was conducted using murine macrophage-like RAW 264.7 cells treated with *P*. *jamasakura* ethanolic and methanolic bark extracts at concentrations of 0, 6.25, 12.5, 25, 50, 100, and 200 *μ*g/ml. For hepatotoxicity assessment, zebrafish larvae were exposed to *P*. *jamasakura* bark extracts, 0.05% dimethyl sulfoxide as a negative control, and 5 *μ*M tamoxifen as a positive control. The surviving larvae were anesthetized and assessed for hepatocyte apoptosis. *Results*. TRAP activity was significantly inhibited (*p* < 0.001) at all concentrations of *P*. *jamasakura* extracts compared to the control treatment. At 50 *μ*g/ml, both ethanolic and methanolic extracts of *P*. *jamasakura* exhibited significant (*p* < 0.01) BMM cell viability compared to the control treatment. *P*. *jamasakura* ethanolic and methanolic extracts had significant inhibitory (*p* < 0.01) effects on lipopolysaccharide (LPS)-induced NO production at 200 *μ*g/ml and exhibited significant (*p* < 0.01) and (*p* < 0.05) stimulative effects, respectively, on RAW 264.7 cell viability. No overt hepatotoxicity was observed in the liver of zebrafish larvae in any of the treatments. *Conclusion*. The TRAP activity of *P*. *jamasakura* bark gives a foundation for further studies to enhance future development of antiosteoporosis drug.

## 1. Introduction

Osteoporosis is a major global public health problem characterized by low bone mass and a deterioration of bone microarchitecture [1]. People suffering from an osteoporosis have increased risk of fractures [2, 3]. It is one of the major causes of morbidity in older people [4] due to imbalance between the bone formation and resorption rate [3]. Several factors have been associated with an increased risk of osteoporosis, including menopause, sex steroid deficiency, and aging [2, 3]. Chronic inflammation has long been associated with a broad range of noninfectious diseases [5], and recent studies suggest that inflammation is one of the key factors that influence bone turnover, leading to osteoporosis [4, 6]. In fact, proinflammatory cytokines have been implicated as primary mediators of accelerated bone loss during menopause [7]. Currently, the treatment of osteoporosis focuses on inhibition of bone resorption by osteoclasts and/or increase in bone formation by osteoblasts [1]. A number of conventional treatment options for osteoporosis are available such as bisphosphonates and estrogen but their adverse effects including burning sensation and gastrointestinal tract disturbances associated with these therapies limit their use [8]. Consequently, exploring the use of natural products in the treatment of osteoporosis may offer a better alternative to avoid the side effects of the conventional therapies [8]. Over the years, herbal medicines have been used to treat osteoporosis [1, 9] and as a crucial substitute of anti-inflammatory drugs [10]. The plants used in traditional medicine for the treatment of inflammation- and osteoporosis-related conditions are those of *Prunus* (family Rosaceae), including *Prunus jamasakura* f. hortensis (Maxim.) (Koidz) (Scientific synonym *Prunus x lannesiana* (Carrière) E. H. Wilson) [11]. *P*. *jamasakura* is native to Korea and Japan and has been used to treat several diseases in folk medicine including inflammatory diseases [12], cough, and food poisoning [11]. These medicinal activities have been attributed to several compounds including sakuranetin, sakuranin, naringenin, and genistein, found in its stem bark (Pruni cortex) [11].

Despite its myriad therapeutic uses, there are currently no studies on the antiosteoporosis activity of *P*. *jamasakura*. Hence, this study evaluated the *in vitro* antiosteoporosis activity of the ethanolic and methanolic bark extracts of *P*. *jamasakura*. In addition, we evaluated the hepatotoxicity of the extracts in zebrafish (*Danio rerio*) larvae and conducted the HPLC chemical profiling of the compounds in the extracts. This study may therefore provide the foundation for further studies regarding *P*. *jamasakura* for future drug development to treat and manage osteoporosis.

## 2. Materials and Methods

### 2.1. Chemicals

All of the chemicals and solvents used in this study were of analytical grade. Acetonitrile (Fisher Scientific, UK) and trifluoroacetic acid (Sigma-Aldrich, USA) were of HPLC grade. Ultrapure water from a Milli-Q system (Millipore, USA) was used for the mobile phase preparation. Naringenin, genistein, and sakuranetin were purchased from ChemFaces (Wuhan, Hubei, China) and were used as the standard components.

### 2.2. Plant Material and Preparation of Extract

The stem bark of *P*. *jamasakura* (Pruni cortex) was procured from Daejeon, South Korea. The voucher specimen number KIOM201501013821A was deposited in the Korean Herbarium of Standard Herbal Resources (Index Herbarium Code: KIOM) at the Korea Institute of Oriental Medicine (KIOM), South Korea. The stem bark of the sample was ground using a steel pulverizing machine (250G New Type Pulverizing Machine, Model RT-N04-2V, Taiwan) at 25,000 rpm to obtain a fine powder. The maceration and concentration process was done following the previous method [13]. 30 g of the fine powder was extracted via maceration using 600 ml of 100% methanol, 100% ethanol, and distilled water. The extracts were filtered using Whatman filter no. 1 after 24 h and concentrated under a vacuum reduced pressure at 40°C, 70 rpm, using an EYELA N-1200B (Tokyo Rikakikai Co. Ltd., Japan) efficient rotary evaporator. The concentrated extract was then vacuum dried. The resultant dried extract was used for subsequent HPLC phytochemical analysis, nitric oxide (NO) assay, tartrate-resistant acid phosphatase (TRAP) assay, and hepatotoxicity evaluation.

### 2.3. HPLC Chemical Profiles of *Prunus jamasakura*

The method used was modified from that of the previous study [13]. The chemical standards naringenin, genistein, and sakuranetin used in this study were each dissolved in methanol at 1 mg/ml to make a stock solution and then further diluted to 20 *μ*g/ml in methanol for the HPLC analysis. Similarly, *P*. *jamasakura* extracts were dissolved in methanol at 10 mg/ml and filtered using a 0.2 mm syringe membrane filter (Whatman Ltd., Maidstone, UK) for analysis. Separation was performed using an HPLC system (Dionex Ultimate 3000; Thermo Fisher Scientific, Sunnyvale, CA, USA) comprising a pump, an auto sampler, a column oven, and a diode array UV/VIS detector. The chromatograms were analyzed using the Chromeleon software system (version 7). The components of the *P*. *jamasakura* extracts were separated using a Gemini C_18_ column (4.6 × 250 mm, 5 *μ*m) (Phenomenex, Torrance, CA, USA) at 40°C. An injection volume of 10 *μ*l was used at a detection wavelength of 280 nm. The mobile phase, consisting of ultrapure water with 0.1% trifluoroacetic acid (A) and acetonitrile (B), was eluted at a flow rate of 1.0 ml/min. The gradient elution program used was as follows: 3% (*v*/*v*) B at 0–2 min; 3–35% (B) at 2–30 min; 35–50% (B) at 30–31 min; 50% (B) at 31–35 min; 50–100% (B) at 35–40 min; and 100% (B) at 40–45 min.

### 2.4. Inhibitory Effect of *P*. *jamasakura* on No Generation and Osteoclastogenesis

#### 2.4.1. Cell Culture

Murine macrophage-like RAW 264.7 cells (ATCC; Manassas, VA, USA) were cultured in Dulbecco's modified Eagle medium supplemented with 10% fetal bovine serum (FBS) and 1% antibiotics following previously described method [13]. Mouse bone marrow macrophages (BMMs) were cultured in a proliferation medium (an *α*-MEM medium with 10% FBS and macrophage-colony stimulating factor (M-CSF) (60 ng/ml)) following the previously described method [14, 15]. To differentiate the osteoclasts, BMMs were cultured in a proliferation medium with RANKL (100 ng/ml) for 6 days.

#### 2.4.2. NO Assay

Murine macrophage-like RAW 264.7 cells were treated with the ethanolic and methanolic extracts of *P*. *jamasakura* samples at various concentrations of 0, 12.5, 25, 50, 100, and 200 *μ*g/ml and cultured for 1 h prior to lipopolysaccharide (LPS) stimulation for 24 h following a previously described method [13]. The nitrite levels in the culture media were determined by incubation with Griess reagent (1% sulfanilamide, 0.1% naphthylethylenediamine dihydrochloride, and 2.5% phosphoric acid) for 5 min. The absorbance was measured at 570 nm using a microplate reader (VersaMax, Molecular Devices). The quantity of nitrite in the samples was calculated using the concentration of sodium nitrite as a standard.

Cell viability was analyzed using a cell counting kit assay (Dojindo). Cells were plated in a 96-well plate and treated with different concentrations of *P*. *jamasakura* extracts at concentrations of 0, 12.5, 25, 50, 100, and 200 *μ*g/ml for ethanolic and methanolic extracts for 24 h. After incubating with the cell counting kit (CCK) solutions and the cells for 1 h, the absorbance was measured at 450 nm using a microplate reader (VersaMax). The results are presented as a percentage of the control.

#### 2.4.3. TRAP Assay and BMM Cell Viability

The measurement of osteoclast TRAP activity was based on the generation of absorbance by incubating BMM cells with TRAP buffer (50 mM sodium tartrate, 0.12 M sodium acetate, and pH 5.2) and *p*-nitrophenyl phosphate (1 mg/ml) for 15 min based on method previously described [14]. For TRAP staining, the cells were incubated with TRAP buffer containing naphthol AS-MX phosphate (0.1 mg/ml) and Fast Red Violet (0.5 mg/ml). The BMM cells were then cultured with the different ethanolic and methanolic *P*. *jamasakura* extracts at different concentrations of 0, 6.25, 12.5, 25, and 50 *μ*g/ml in the presence of RANKL for 6 days. The osteoclast TRAP activity was determined using a colorimetric assay with *p-*nitrophenyl phosphate as a substrate. The cell viability was determined using cell counting kit-8 (WST-8/CCK8; Dojindo), according to the manufacturer's instructions.

For the measurement of cell viability, cells were plated in 96-well plates and treated with ethanolic and methanolic *P*. *jamasakura* extracts at different concentrations of 0, 6.25, 12.5, 25, and 50 *μ*g/ml for 24 h. After incubating with the CCK solutions and the cells for 1 h, the absorbance was measured at 450 nm using a microplate reader (Versa Max). The results are presented as a percentage of the control.

### 2.5. Hepatotoxicity Assay in Zebrafish (*Danio rerio*) Larvae

Zebrafish larvae were bred under standard conditions as previously described [16] (Westerfield, 2000). At 90 h postfertilization (hpf), the larvae were transferred to a transparent 24-well plate (*N* = 10/well) with 1 ml of an embryonic medium. The larvae were then exposed to water, ethanolic, and methanolic *P*. *jamasakura* extracts at various concentrations of 50, 100, and 200 *μ*g/ml for ethanolic and methanolic, and water extracts from 96 to 120 hpf. Dimethyl sulfoxide (DMSO) was used as a negative control while 5 *μ*M of tamoxifen (Sigma-Aldrich, St. Louis, MO, USA) was used as a positive control. To obtain images, the larvae were anesthetized in tricaine (Sigma-Aldrich), mounted in 3% methyl cellulose (Sigma-Aldrich), and observed under a Leica MZ10F stereomicroscope equipped with a Leica DFC425 camera and Leica application Suite software (version 4.5).

### 2.6. Statistical Analysis

Data are represented as the mean ± standard deviation. Statistical significance between groups was analyzed using Student's *t*-test. *p* values <0.05 were considered statistically significant.

## 3. Results and Discussion

### 3.1. HPLC Chemical Profiles of *Prunus jamasakura*

**Figure 1:**
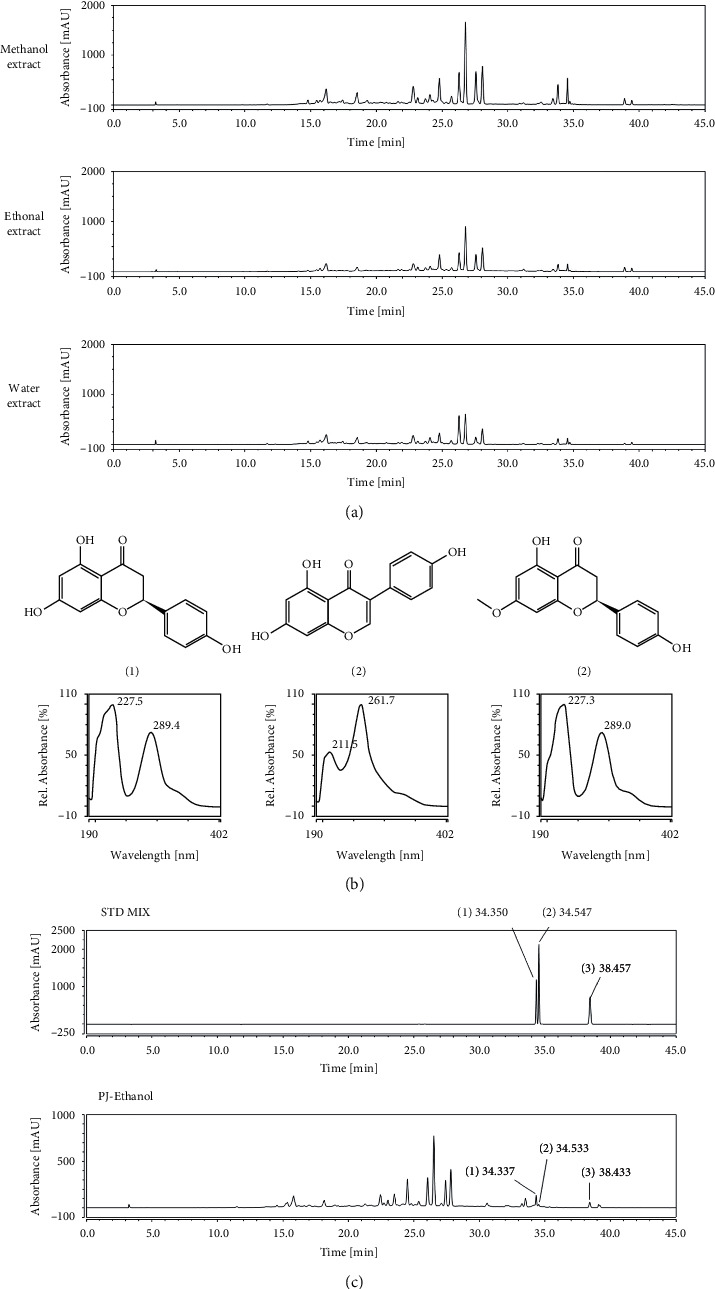
HPLC-DAD analysis of phytochemical components of *P*. *jamasakura*. (a) HPLC fingerprint of the ethanolic, methanolic, and water extracts of *P*. *jamasakura* at 280 nm. (b) Chemical structures and UV spectrum of three standard components: (1) naringenin, (2) genistein, and (3) sakuranetin. (c) Determination of naringenin (1), genistein (2), and sakuranetin (3), in *P*. *jamasakura* ethanol extracts at 280 nm.

HPLC is a versatile, reproducible chromatographic technique for the estimation and detection of secondary metabolites in plants [17]. In this study, the phytochemical components of *P*. *jamasakura* based on the HPLC fingerprinting of their methanol, ethanol, and water extracts at 203, 254, 280, and 320 nm UV wavelengths (data not shown) were conducted. Among the four types of UV wavelengths, good separation and selectivity were observed at 280 nm. The distinct profiling patterns of the components were confirmed regardless of the type of solvent used in Figure 1(a).

Three chemical compounds, namely, naringenin [18], genistein [19], and sakuranetin [1], were selected as the standard components of *P*. *jamasakura*, according to a previous study [11]. The UV wavelength of the chromatograms was adjusted to 280 nm according to the maximum UV absorption of the three standard components (Figure 1(b)). The identification of the three standard components in *P*. *jamasakura* was based on a comparison between their retention times (*t*_*R*_), UV absorption, and chromatograms and those of each standard. The mixed standard components were identified at retention times of 34.350 min (1), 34.547 min (2), and 38.457 min (3) in the chromatogram. Under the same conditions, three component standards were detected at similar retention times, 34.337 min (1), 34.533 min (2), and 38.433 min (3) in the *P*. *jamasakura* ethanol extract (Figure 1(c)). Therefore, this HPLC result showed the presence of naringenin, genistein, and sakuranetin compounds in the stem bark of *P*. *jamasakura.*

### 3.2. NO Assay and RAW264.7 Cell Viability

**Figure 2:**
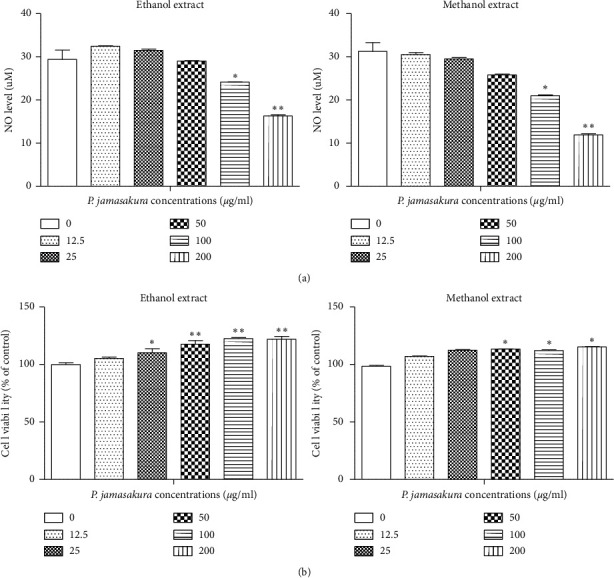
Effect of *P*. *jamasakura* on LPS-induced NO production in raw 264.7 cells. RAW264.7 cells were pretreated with *P*. *jamasakura* for 1 h before LPS treatment. (a) After 24 h incubation with LPS, the culture supernatant was collected for measurement of nitrite concentration. (b) Cell viability was determined using cell counting kit-8 following the manufacturer's instructions. ^*∗*^*p* < 0.05, ^*∗∗*^*p* < 0.01, and ^*∗∗∗*^*p* < 0.001.

NO plays a key role in the immune system defense against extracellular organisms [20]. However, when produced in excess, it is known to play an important role in the pathogenesis of inflammatory disorders of the joint, gut, and lungs [13, 21, 22]. A previous study also showed that increased NO production contributes to the pathogenesis of osteoporosis [23]. Therefore, the inhibition of NO production represents a potential therapeutic pathway for the management of inflammatory diseases [21]. In this study, the *P*. *jamasakura* extracts exhibited inhibitory effects on NO production (Figure 2). At 100 *μ*g/ml and 200 *μ*g/ml, the ethanolic and methanolic *P*. *jamasakura* extracts showed significant (*p* < 0.05) and (*p* < 0.01) inhibition of the LPS-induced NO production in RAW264.7 cells, respectively, compared to the control (Figure 2(a)). At concentrations of 50, 100, and 200 *μ*g/ml, the ethanolic and methanolic extracts exhibited significant cell (*p* < 0.001) and (*p* < 0.05) simulative effect, respectively, on cell viability (Figure 2(b)).

A previous study reported the downregulation of NO production by sakuranetin and naringenin compounds [12], and hence the presence of these compounds in *P*. *jamasakura* may somewhat explain its ability to inhibit LPS-mediated NO production in RAW 264.7 cells in this study.

### 3.3. TRAP Assay and BMM Viability

**Figure 3:**
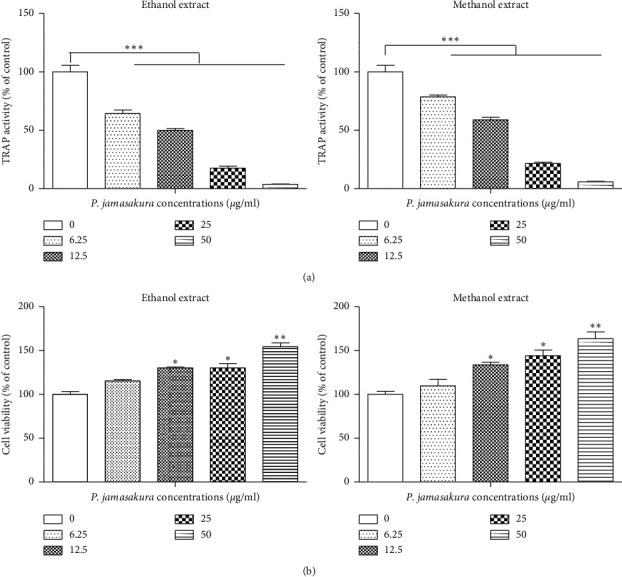
Effect of *P*. *jamasakura* on trap activity in BMM. The BMM was cultured with *P*. *jamasakura* in the presence of RANKL for 6 days. (a) TRAP activity of osteoclasts was measured by colorimetric assay using *p*-nitrophenyl phosphate as a substrate. (b) Cell viability was determined using cell counting kit-8 following the manufacturer's instructions. ^*∗*^*p* < 0.05, ^*∗∗*^*p* < 0.01, and ^*∗∗∗*^*p* < 0.001.

TRAP activity is a vital cytochemical marker of osteoclasts. As such, its concentration in the serum is utilized as a biochemical and histochemical marker of osteoclast function and degree of bone resorption [24, 25]. A previous study showed that the levels of serum TRAP were higher in patients with osteoporosis than in the control group. Furthermore, a negative linear correlation was previously found between serum TRAP and bone mineral content in women with osteoporosis, suggesting that TRAP concentration is a useful marker for bone loss [26]. Indeed, all forms of acquired osteoporosis reflect increased osteoclast function relative to that of osteoblasts, such that the pharmacological arrest of osteoclasts is a mainstay in the treatment of systemic bone loss [27]. TRAP initiates osteoclast differentiation, activation, and proliferation [18] and has been observed to play a vital role in many biological processes, including skeletal development, collagen synthesis and degradation, and bone mineralization [25, 28]. In this study, the ethanolic and methanolic *P*. *jamasakura* extracts at different concentrations of 0, 6.25, 12.5, 25, and 50 *μ*g/ml all significantly (*p* < 0.001) inhibited TRAP activity compared to the control (Figure 3(a)).

At 12.5 and 25 *μ*g/ml, *P*. *jamasakura* methanolic and ethanolic extracts had a significant effect (*p* < 0.05) simulative effect on the cell viability of BMM cells (Figure 3(b)). At a higher concentration of 50 *μ*g/ml for both ethanolic and methanolic extracts, the cell viability increased significantly (*p* < 0.01) compared to the control. The significant viability of the BMM cells treated with all provides an indication of the noncytotoxicity of the stem bark of *P*. *jamasakura* within a given dose range.

The suppression of TRAP activity by *P*. *jamasakura* may also be attributed to the various chemicals in it including naringenin and genistein. Naringenin has been observed to inhibit osteoclastogenesis and osteoclastic bone resorption [29, 30]. Genistein also showed direct inhibitory effect on osteoclasts *in vitro* [31] and suppressed TRAP activity [32]. Previous studies have also shown the antiosteoporosis activity of other *Prunus* species [33, 34]. The bioactive compounds in the fruit of *P*. *mume* were found to significantly stimulate the differentiation of preosteoblastic MC3T3-E1 cells to increase collagen synthesis or mineralization functions of osteoblasts and suppress TRAP activity in the receptor activator of nuclear factor-*κ*B ligand-induced osteoclastic RAW 264.7 cells [35]. Additionally, terpenes and sterols in the fruit of *P*. *mume* were found to inhibit osteoclast differentiation by suppressing TRAP activity [36]. These results further validates the potential of *Prunus* species as an antiosteoporosis medicinal plant.

### 3.4. Hepatotoxicity in Zebrafish Larvae

**Figure 4:**
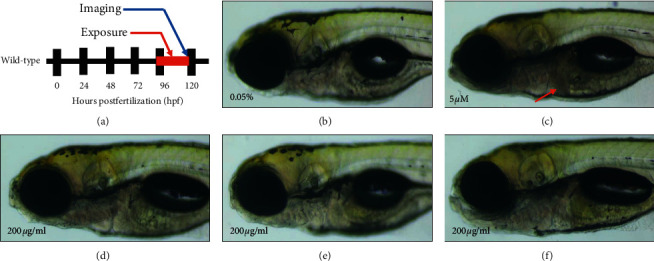
Hepatotoxicity assay in zebrafish larvae. (a) Schematic diagram of hepatotoxicity assay in zebrafish. (b) Treatment with DMSO did not show liver cell death. (c) Tamoxifen treatment induced liver cell death (red arrow). (d) No liver cell death was observed at concentration of 200 *μ*g/ml of water extract. (e) No liver cell death was observed at concentration of 200 *μ*g/ml of ethanolic extract. (f) No liver cell death was observed at concentration of 200 *μ*g/ml of methanolic extract.

The hepatotoxicity results for a given drug are vital for understanding its potential effects on the liver and the potential induction of liver injury [37]. Herbal medicines have been previously associated with various complications, such as liver damage, which result in high incidences of mortalities and morbidities [19]. Hence, the screening of herbal medicines for their hepatotoxicity is a key step during the development of herbal medicines. Zebrafish larvae represent an important model system for the study of the effects of toxicant exposure on liver function and development [38, 39], as well as changes in red fluorescence intensity and size, making it a useful model for hepatotoxicity studies [40]. Liver organogenesis in zebrafish initiates at 30 hpf on the left-hand side of the embryo. In this study, the liver bud was enlarged, connected with the intestine, and functionally matured until 72 hpf [41]. At 96 hpf, treatment with liver toxicants (tamoxifen or acetaminophen) induced a reduction in liver transparency, indicating liver cell death in the zebrafish larvae [42]. In this study, the exposure of the zebrafish larvae from 96 to 120 hpf (Figure 4(a)) to DMSO did not result in the liver cell death (Figure 4(b)). However, treatment with tamoxifen resulted in liver cell death (Figure 4(c)). In *P*. *jamasakura* bark water and ethanolic extract at the concentrations of 50, 100, and 200 *μ*g/ml, 100% of the zebrafish larvae survived at 120 hpf and hepatocyte death was not observed. Similarly, there was 100% survival of the zebrafish larvae exposed to *P*. *jamasakura* bark methanolic extract at concentrations at 50 *μ*g/ml with no hepatocyte death observed at 120 hpf (Figures 4(d) and 4(e)). However, at higher concentrations of 100 and 200 *μ*g/ml *P*. *jamasakura* bark methanolic extract, only 30% and 20% of the zebrafish larvae, respectively, survived but hepatocyte death was not observed in them (Figure 4(f)).

Hepatotoxicity observed in the zebrafish larvae model system correlates with that in humans [43], an indication that *P*. *jamasakura* extracts may have a low hepatotoxic effect on other vertebrates, including humans.

## 4. Conclusion

The use of medicinal plants to treat myriad of conditions including osteoporosis and inflammation has been in existence since time immemorial. This present study has demonstrated the antiosteoporosis, anti-inflammatory, and nonhepatotoxic activities of *P*. *jamasakura.* And as such, the study provides a basis for clinical studies and a foundation upon which *P*. *jamasakura* antiosteoporosis drugs can be developed in the future.

## Data Availability

The raw data supporting the results of this article will be made available through the corresponding author upon request.

## Abbreviations

BMMs: Bone marrow macrophages
CCK: Cell counting kit
FBS: Fetal bovine serum
HPLC: High-performance liquid chromatography
LPS: Lipopolysaccharide
M-CSF: Macrophage-colony stimulating factor
NO: Nitric oxide
TRAP: Tartrate-resistant acid phosphatase.
